# 3D6 and 4B3: Recombinant expression of two anti-gp41 antibodies as dimeric and secretory IgA

**DOI:** 10.1186/1753-6561-5-S8-P56

**Published:** 2011-11-22

**Authors:** David Reinhart, Robert Weik, Renate Kunert

**Affiliations:** 1 Department of Biotechnology, Institute of Applied Microbiology, University of Natural Resources and Life Sciences, Vienna, Austria; 2 Polymun Scientific Immunbiologische Forschung GmbH, Vienna, Austria

## Background

Sexually transmitted diseases are predominantly acquired via mucosal membranes of the rectal or genital tract during sexual intercourse. This major port of virus entry is naturally defended by the humoral immune response, with immunoglobulin A (IgA) as the primary antibody class to elicit mucosal immunity. Dimeric IgA (dIgA) reaches the luminal side of mucosal tissues by transcytosis through epithelial cells lining the mucosa. In a first step, dIgAs specifically bind to the basolaterally expressed polymeric immunoglobulin receptor (pIgR) on epithelial cells. For release of IgAs on the luminal side the extracellular portion, termed secretory component (SC), remains attached to the antibody to form secretory IgA (sIgA) [1,2].

One example of a sexually transmitted disease is the human immunodeficiency virus (HIV) which annually infects several million individuals on a global scale and potentially leads to the acquired immunodeficiency syndrome (AIDS). Although current therapies can reduce disease progression in infected individuals, no cure is yet available or within reach in near future. As a consequence increased attention is now being paid to develop drugs that could prevent virus acquisition.

3D6 and 4B3 are two monoclonal antibodies (mAb) which have originally been isolated as IgG1 isotype from seroconverted HIV-1 patients and bind to the principal immunodominant domain of gp41. In the course of this project both mAbs were isotype switched to IgA1. Recombinant CHO cell lines were established for the production of 3D6 and 4B3 as dimeric as well as secretory IgA. While dIgA were expressed by a single cell line, sIgA are produced by a biochemical association of dIgA with SC. Both dIgA and sIgA variants were characterized and the contribution of the heavily glycosylated SC on IgA stability will be investigated.

## Antibody expression

MAbs 3D6 and 4B3 (IgG1) were developed at the Institute of Applied Microbiology [3,4]. Isotype switching was performed by substitution of the original heavy chain constant region with that for IgA1 (GenBank Accession Number 184743). For their recombinant expression as dIgA three plasmids were generated containing the coding region of either heavy chain, light chain or joining (J) chain. The latter one should increase dimerization of IgA monomers. Recombinant CHO clones were selected whereas high producers were isolated by applying the dihydrofolate reductase (dhfr) system.

Assembly of 3D6 and 4B3 as sIgA is intended by an *in vitro* biochemical association of dimeric IgA with human secretory component (hSC). Thus, CHO cell lines which solely express hSC were established by co-transfection of two plasmids containing the coding region for hSC and dhfr, respectiviely.

## Cultivation temperature greatly influences productivity

Mammalian cells are commonly cultivated at 37°C. In order to investigate the effect on mAb secretion, clones expressing 3D6-dIgA and 4B3-dIgA were propagated at sub-physiological temperatures. It was observed that temperature markedly impacts final IgA quantities in culture supernatants. Product titers of clones expressing 3D6-dIgA were almost 3-fold increased when incubated at 33 °C as compared to 37 °C. Furthermore, cultivation of 4B3-dIgA-expressing clones at 33 °C, rather than at 37 °C, resulted in end titers which were approximately 13-fold increased.

## Purification of recombinant dIgA

The two established mAb-expressing cell lines secrete dIgA at different quantities: The best clone producing 3D6-dIgA achieved a mean specific productivity of 59.1±14 pg/cell*day (pcd). Conversely, the highest 4B3-dIgA secreting clone reaches a mean specific productivity of 0.6±0.4 pcd. Hence, an increased amount of host cell proteins is present in cell culture supernatants of 4B3-dIgA as compared to 3D6-dIgA. Therefore, we elaborated a purification protocol which allows the recovery of both dimeric IgAs at a greatly enhanced purity. The purification procedure is shown in table 1.

**Table 1:** Purification scheme developed for isolation of 3D6 and 4B3 dIgA.

STEP	EQUIPMENT	PROCEDURE	RECOVERY	
			3D6 dIgA	4B3 dIgA
Ultra/Diafiltration	Kvick Start UDF Cassette, Millipore, 30 kD	Harvested cell culture supernatant containing dIgA is concentrated and buffer exchanged against PBS, pH 7.4	>95 %	>95 %
Lectin Affinity Chromatography	Immobilized Jacalin, Thermo Scientific	UDF retentate is immobilized onto a lectin affinity resin. After a washing step, bound product is eluted by applying 1.5 M D-galactose in PBS, pH 7.4	98.7 %	106.1 %
Ultra/Diafiltration	Kvick Start UDF Cassette, Millipore, 30 kD	The eluate is buffer exchanged against 20 mM Tris, 10 mM NaCl, pH 8.5	>95 %	>95 %
Anion Exchange Chromatography	DEAE Sepharose FF, GE Healthcare	The retentate is applied onto the anion exchanger. Post washing with 100mM NaCl product is eluted from the resin using 20 mM Tris, 200 mM NaCl, pH 8.5	88.4 %	86.7 %
Hydrophobic Interaction Chromatography	Phenyl-Sepharose 6FF low sub, GE Healthcare	(NH_4_)_2_SO_4_ is added to 0.75 M for 4B3-dIgA or 1.25 M for 3D6-dIgA to precipitate host cell proteins but not mAb itself. After a washing step 3D6-dIgA is eluted with 0.4 M (NH_4_)_2_SO_4_, which allows to isolate dimeric IgA from other IgA isoforms. 4B3-dIgA desorbs at 0 M (NH_4_)_2_SO_4_	44.1 %	59.1 %

## Formation of secretory IgA

Secretory IgA can be assembled *in vitro* due to the natural affinity of dimeric IgA for secretory component and *vice versa* (5). Initially, supernatants from recombinant cell lines expressing hSC, 3D6-dIgA or 4B3-dIgA were buffer exchanged against PBS. Subsequently, sIgA formation was performed by incubation of hSC with dIgA at 25°C for 3 hours at different molar ratios. However, applying a molar ratio of 1:1 is commonly described as optimal [5]. The association of 3D6-dIgA or 4B3-dIgA with hSC was successfully verified by SDS-PAGE following Western blotting (data not shown). Conjugation of the immunoblot was performed with a mouse anti-human secretory component antiserum (Sigma) and detected via an anti-mouse IgG1 antiserum (Sigma).

## Conclusions

The anti-HIV-1 mAbs 3D6 and 4B3 were isotype switched and could successfully be expressed as dIgA in stably transfected CHO cells. Furthermore, continuous product secretion was obtained for at least 20 passages in spinner flasks.

Cultivation of the recombinant cell lines at different temperatures revealed that their product expression can markedly be influenced: 3D6-dIgA expression was nearly triplicated by shifting the temperature from 37 °C to 33 °C. Applying the same conditions, 4B3-dIgA product secretion was nearly 13-fold increased.

A purification protocol was developed which allows the recovery of the dIgAs 3D6 and 4B3 in highly pure fractions. Furthermore, the elaborated scheme enables the isolation of dimeric 3D6 from its various high molecular weight isotypes.

Secretory IgA of both 3D6 and 4B3 can successfully be produced by mixing dimeric IgA with human secretory component.
